# Velocity Estimation of Passive Target Based on Sparse Bayesian Learning Cross-Spectrum

**DOI:** 10.3390/s24216989

**Published:** 2024-10-30

**Authors:** Xionghui Li, Guolong Liang, Tongsheng Shen, Zailei Luo

**Affiliations:** 1 College of Underwater Acoustic Engineering, Harbin Engineering University, Harbin 150001, China; wananmotianlun@foxmail.com (X.L.); liangguolong@hrbeu.edu.cn (G.L.); 2 Advanced Interdisciplinary Technology Research Center, National Innovation Institute of Defense Technology, Beijing 100071, China; shents_bj@126.com; 3 Key Laboratory of Marine Information Acquisition and Security, Harbin Engineering University, Harbin 150001, China; 4 National Key Laboratory of Underwater Acoustic Technology, Harbin Engineering University, Harbin 150001, China

**Keywords:** sparse Bayesian learning, cross-spectrum, velocity estimation

## Abstract

To solve the poor performance or even failure of the cross-spectrum (CS) method in hydroacoustic weak-target passive velocimetry, a sparse Bayesian learning cross-spectrum method (SBL-CS), combining phase compensation with sparse Bayesian learning (SBL) is proposed in this paper. Firstly, the cross-correlation sound intensity is taken as the observation quantity and compensates for each frequency point of the cross-spectrum, which enables the alignment of cross-spectrum results at different frequencies. Then, the inter-correlation sound intensity of all frequencies is fused in the iterative estimation of the target velocity, verifying the proposed method’s ability to suppress the background noise when performing multi-frequency processing. The simulation results show that the proposed method is still effective in estimating the target velocity when the CS method fails and that the performance of the proposed method is better than the CS method with a decrease in SNR. As verified using the SWellEx-96 sea trial dataset, the RMSE of the proposed method for surface vessel speed measurement is 0.3545 m/s, which is 46.1% less than the traditional CS method, proving the feasibility and effectiveness of the proposed SBL-CS method for the estimation of the radial speed of a passive target.

## 1. Introduction

The motion velocity estimation of passive targets is a trending and complex research topic in the field of hydroacoustics [1,2,3]. The distance–frequency interference structure phenomenon in the acoustic field is an important feature of the underwater waveguide [4,5,6,7], which can reflect the target’s depth, distance, and velocity. The target’s velocity information is estimated by the interferometric structure of the interrelated field [8], which is related to the amount of target distance variation but is not sensitive to the distance itself. Therefore, the cross-spectrum method has a low dependence on external environmental parameters and is suitable for target velocity estimation using a single hydrophone.

The cross-spectrum method [8,9], as one of the commonly used target velocity estimation methods, has gained the attention of many researchers and has been used in many scientific studies. Sun [10] extended the mutual spectrum method to different seabed environments and analyzed the performance of the mutual spectrum method. Yang et al. [11] analyzed the broadband field interference characteristics of the mutual spectrum method and proposed a target radial velocity estimation method and a sound source depth estimation method, based on the use of deep-sea-sitting single hydrophones. Aiming for the mutual spectrum method to fail under low signal-to-noise ratio conditions, Zhao et al. [12] used phase compensation and histogram estimation of line spectrum frequency points to realize the fusion of the velocity estimation results for multi-frequency points. To address the estimation error introduced by the histogram grid width, Song [13] combined an equalization window with a reciprocal spectrum histogram, which further suppressed the noise interference and improved the speed measurement accuracy. However, under the condition of the number of points being constant, the lower the frequency selected for processing, the lower the spectral resolution of the cross-spectrum method, which can also affect the speed measurement accuracy. At the same time, low-frequency signals are susceptible to environmental noise interference in actual marine environments. The CS algorithm cannot directly accumulate the processing results of different frequencies, resulting in the algorithm being unable to obtain more gain and the single line spectrum being easily overwhelmed by noise, leading to algorithm failure. Therefore, the accuracy of the CS algorithm is poor or even ineffective under low signal-to-noise ratio conditions. Research on passive target velocity measurement algorithms that can operate under low signal-to-noise ratio conditions is needed.

Sparse decomposition theory was first proposed in the 1990s [14] and its principle is based on the sparse representation of signals, which uses less information to represent the characteristics of signals. Sparse Bayesian learning (SBL) uses a Bayesian framework to find sparse solutions to underdetermined linear problems [15]. Since SBL can automatically determine sparsity [16,17,18], it has been widely used in high-resolution orientation estimation [19,20,21,22]. In order to solve the problem of methodic failure of the cross-spectrum method under low signal-to-noise ratio conditions, this paper fuses SBL with the cross-spectrum method, improves it, and proposes the Sparse Bayesian learning cross-spectrum (SBL-CS) method.

The proposed method realizes the accumulation of the results of different frequency cross-spectra by compensating all frequency points of the cross-spectrum. During the iteration process, the method fuses the results of all frequency points solved and estimates the target velocity to achieve noise suppression. The paper is organized as follows: Section 2 derives the speed measurement model under simple normal wave theory and the SBL-CS method. Section 3 compares the SBL-CS method with the mutual spectrum method, using simulation experiments, and verifies the effectiveness of the SBL-CS method using a real dataset. Section 4 summarizes the whole paper.

## 2. Sparse Bayesian Learning Mutual Spectrum Velocimetry

### 2.1. Modeling of Cross-Correlating Sound Fields

The pressure fields at a distant range *r* and the depth $z_{r}$ from a radiating source are denoted as $p(r,z_{r})$. According to normal mode theory, the source signal frequency is *f*, and the depth of source is *z*. Also according to normal mode theory, the expression of the sound field is as follows [23,24]:

(1) $$p\left(r,z_{r}\right)≃\frac{i}{\rho \left(z\right)\sqrt{8\pi r}}e^{-i\pi /4}\sum_{m=1}^{M}\psi_{m}\left(z_{r}\right)\psi_{m}\left(z\right)\frac{e^{ik_{rm}r}}{\sqrt{k_{rm}}}$$

where $\psi_{m}\left(z\right)$ is the amplitude of the *m*th normal mode, $k_{rm}$ is the horizontal wavenumber of the *m*th normal mode, ρ(z) is the density at the sound source, and *i* is the imaginary unit.

Assuming the target maintains the same depth *z* and moves with a uniform velocity in the horizontal plane at $v_{0}$, the velocity of the target, the radial velocity in the horizontal direction relative to the hydrophone is $v_{r}$,meaning that the expression of the sound field at the distance r+Δr is as follows [7]:

(2) $$\begin{array}{c} p\left(r+\Delta r,z_{r}\right)≃\frac{i}{\rho \left(z\right)\sqrt{8\pi (r+\Delta r)}}e^{-i\pi /4}\sum_{m=1}^{M}\psi_{m}\left(z_{r}\right)\psi_{m}\left(z\right)\frac{e^{ik_{rm}\left(r+\Delta r\right)}}{\sqrt{k_{rm}}} \end{array}$$

Due to the slow movement of the target in the real environment, r≫Δr, we have 11r+Δrr+Δr≈11rr. Setting $C=1/8\pi r\rho {\left(z\right)}^{2}$ and Am=ψm(zr)ψm(z)ψm(zr)ψm(z)krmkrm, as well as cross-correlating the pressure signals received at distances *r* and r+Δr, yields the following equation [2]:

(3) $$\begin{array}{c} I_{r,r+\Delta r}\left(\Delta r\right)=p\left(r+\Delta r,z_{r}\right)p*\left(r,z_{r}\right)\approx C\sum_{m,n}A_{m}A_{n}e^{-j\Delta k_{mn}\bar{r}}e^{j\bar{k_{r}}\Delta r} \end{array}$$

where overliner=r+ΔrΔr22, $\bar{k_{r}}$ is the mean horizontal beam, kr¯=2πf2πfcpcp, $c_{p}$ is the mean phase velocity, and $\Delta k_{mn}=k_{rm}-k_{rn}$.

Since r≫Δr, the change of $\sum_{m,n}A_{m}A_{n}e^{-j\Delta knm\bar{r}}$ in Equation (3) is very slow, making $D=C\sum_{m,n}A_{m}A_{n}e^{-j\Delta knm\bar{r}}$. The distance change is $\Delta r=v_{r}\Delta t$. Substituting this into Equation (3) yields the following:

(4) $$I_{r,r+\Delta r}\left(\Delta t\right)\approx De^{j\bar{k_{r}}\Delta r}=De^{j2\pi fv_{r}\Delta t/c_{p}}$$

From Equation (4), there exists a mapping relationship between the oscillation frequency of the cross-correlating sound intensity $f_{I}$ and the target motion velocity $v_{r}$, as follows:

(5) $$f_{I}=fv_{r}/c_{p}$$

The positive or negative value of $v_{r}$ corresponds to the two states of the target being far away and close to the target, so $f_{I}$ can be positive or negative.

The radial velocity of the target can be measured by finding the frequency of the interlocking sound intensity. However, a change in the signal frequency *f* will cause a change in the oscillation frequency of the cross-correlating sound intensity $f_{I}$; therefore, when there is more than one target line spectrum, the cross-correlating sound intensity spectra obtained from the line spectra cannot be directly summed up. Instead, it must be compensated for additionally.

### 2.2. Joint Multi-Frequency Velocity Estimation Method

When the sound source is a multilinear spectrum, set the line frequency $F=\{f_{1},f_{2},\ldots ,f_{L}\}$. The cross-correlating sound intensity is then written as follows:

(6) Ir,r+Δr(Δt,fl)≈Dflej2πflvrΔt2πflvrΔtcpcp

where $D_{f_{l}}$ is a constant at different line spectral frequencies $f_{l}$ and l=1,2,…,L.

From Equation (4), we can see that the interrelated sound intensity $I_{r+\Delta r}\left(\Delta t,f_{l}\right)$ is a complex signal, and the Fourier transform of the cross-correlating sound intensity is given by the following equation:

(7) YI(n,fl)≈∑i=−NNIr,r+Δr(iΔt,fl)e−j2πin/(N)=Dfl∑i=−NNej2πi(ΔtflvrΔtflvrcpcp−nnNN)

where n=−N,…,−1,0,1,…,N.

If the term $\Delta tf_{l}v_{r}/c_{p}-n/N$ in Equation (7) is minimized by $n_{0}$, then $n_{0}$ is the peak position of the spectrum of the interlocking sound intensity. Let $n_{0,l}$ be the peak position of the frequency domain of the cross-correlating sound intensity $Ir+\Delta r(\Delta t,f_{l})$. Then, this yields the following equation:

(8) $$v_{r}=\frac{n_{o,l}c_{p}}{Nf_{l}\Delta t}$$

From Equation (8), it can be seen that the velocity resolution and spectral peaks are different for different frequencies of the cross-correlating sound intensity. When estimating the frequency of the interlocking sound intensity, phase compensation can be performed according to different frequencies. Let $f_{k}$ be the specified alignment frequency, $n=n^{\prime }f_{l}/f_{k}$. From Equation (7), we can then deduce the following equation:

(9) YI′(n′,fl)≈Dfl∑i=−NNej2πi(ΔtflvrΔtflvrcpcp−n′fln′flfkNfkN)

Referring to Equation (8), it can be deduced that $\forall f_{l}\in F$ are both as follows:

(10) $$v_{r}=\frac{n_{o,l}c_{p}}{Nf_{l}\Delta t}=\frac{{n_{o,l}}^{\prime }c_{p}}{Nf_{k}\Delta t}$$

After phase compensation, the velocity resolution is changed to $c_{p}/Nfk\Delta t$, the upper limit of observable velocities is changed to $c_{p}/fk\Delta t$, and the position of the peaks of the solved spectrum ${n_{0,l}}^{\prime }$ is not affected by the processing frequency $f_{l}$, which is determined by the alignment frequency $f_{k}$.Therefore, the phase-compensated interrelated sound intensity spectra ${Y_{I}}^{\prime }$ can be added directly.

Gain can be obtained by summing the spectra from multiple frequency solvers. Multi-line spectral summing can operate at lower signal-to-noise ratios than relying on a single line running at spectra-solving speed.

### 2.3. Sparse Bayesian Learning Speed Estimation Model

A target corresponds to a velocity, and the velocity corresponds to the vibration frequency of the cross-correlating sound intensity, so the vibration frequency of the cross-correlating sound intensity is sparse in the spectrum. Considering the effect of noise, Equation (6) can be expressed as follows:

(11) $$I\left(f_{l}\right)=A\left(V,f_{l}\right)S\left(f_{l}\right)+N\left(f_{l}\right)$$

where $I\left(f_{l}\right)={\left[I_{1}\left(f_{l}\right),\ldots ,I_{M}\left(f_{l}\right)\right]}^{T}$ is the M × 1-dimensional data vector, $A(V,f_{l})$ is the M × (2N + 1)-dimensional dictionary matrix, $S(f_{l})$ is the (2N + 1) × 1-dimensional target velocity information, and $N(f_{l})$ is the M × 1-dimensional noise matrix.

According to the sparse theory, the target speed $\left[-c_{p}/f_{k}\Delta t,c_{p}/f_{k}\Delta t\right]$ is uniformly divided into (2N + 1) parts, and the over-complete dictionary matrix $A\left(V,f_{l}\right)=[A\left(v_{1},f_{l}\right),\ldots ,$$A(v_{N},f_{l})]$ is obtained, in which the elements of the n column of the dictionary matrix are as follows:

(12) $$a\left(v_{n},f_{l}\right)={\left[1,e^{j2\pi \Delta tf_{l}n/Nf_{k}},\ldots ,e^{j2\pi \left(M-1\right)f_{l}n/Nf_{k}}\right]}^{T}$$

Utilizing the sparse characteristics of the signal in the frequency domain, the hyperparameter $\mathrm{fl}={\left[\gamma_{1},\ldots ,\gamma_{N}\right]}^{T}$ is introduced to perform variational approximation of $S_{f}$, and the target orientation estimation is realized by the alternating iteration of the hyperparameter fl and the noise ${\sigma_{l}}^{2}$.

If the signal is uncorrelated with the noise, the likelihood probability of the data can be written as follows:

(13) $$\begin{array}{c} p\left(I_{f}|S_{f}\right)=\prod_{m=1}^{M}p(I_{m}\left(f_{l}\right)|s_{m}\left(f_{l}\right))=\prod_{m=1}^{M}CN(I_{m}\left(f_{l}\right);A\left(V,f_{l}\right)s_{m}\left(f_{l}\right),{\sigma_{l}}^{2}I) \end{array}$$

Let $\Gamma =diag\left(\gamma_{1},\ldots ,\gamma_{N}\right)=diag\left(\mathrm{fl}\right)$. Then, this yields the following prior model:

(14) $$\begin{array}{c} p\left(S_{f}\right)=\prod_{m=1}^{M}p(s_{m}\left(f_{l}\right))=\prod_{m=1}^{M}CN(s_{m}\left(f_{l}\right);0,\Gamma ) \end{array}$$

Deriving Bayesian evidence from prior and likelihood probabilities under Gaussian assumptions [25,26,27], the following equation is yielded:

(15) $$\begin{array}{c} p\left(I_{f}\right)=\int p\left(S_{f}\right)p\left(I_{f}|S_{f}\right)dS_{f}=\prod_{m=1}^{M}CN(I_{m}\left(f_{l}\right);0;\sum_{I_{m}\left(f_{l}\right)}) \end{array}$$

where $\sum_{I_{m}\left(f_{l}\right)}=\sum_{n_{f}}+A\left(V,f_{l}\right)\Gamma A{\left(V,f_{l}\right)}^{H}$ and $\sum_{n_{f}}{=\sigma_{l}}^{2}I$.

Estimating the hyperparameters fl by maximizing the log Bayesian evidence gives the following equation:

(16) $$\begin{array}{c} \left({\hat{\gamma }}_{1},\ldots ,{\hat{\gamma }}_{N}\right)=\arg\max_{\gamma }\ln p\left(Y_{f}\right)=\arg\max_{\gamma }\left\{-\sum_{m=1}^{M}\left(I_{m}{\left(f_{l}\right)}^{H}\Sigma_{I_{m}\left(f_{l}\right)}^{-1}I_{m}\left(f_{l}\right)+\ln|\Sigma_{I_{m}\left(f_{l}\right)}^{-1}|\right)\right\} \end{array}$$

The hyperparameters fl and $\sum_{I_{m}\left(f_{l}\right)}$ can be updated from the last iteration. The update of γ using the fixed-point iteration method results in the following equation [25,26,27]:

(17) $${\gamma_{n}}^{k+1}={\gamma_{n}}^{k}\left(\frac{\sum_{m=1}^{M}|I_{m}{\left(f_{l}\right)}^{H}\Sigma_{I_{m}\left(f_{l}\right)}^{-1}a\left(v_{n},f_{l}\right){|}^{2}}{\sum_{m=1}^{M}a{\left(v_{n},f_{l}\right)}^{H}\Sigma_{I_{m}\left(f_{l}\right)}^{-1}a\left(v_{n},f_{l}\right)}\right)$$

For different line spectral frequencies of the same target, the spectra of the correlated sound intensities are aligned. Therefore, the iterative formula at multiple frequencies is obtained as follows:

(18) $${\gamma_{n}}^{k+1}={\gamma_{n}}^{k}\left(\frac{\sum_{l=1}^{L}\sum_{m=1}^{M}|I_{m}{\left(f_{l}\right)}^{H}\Sigma_{I_{m}\left(f_{l}\right)}^{-1}a\left(v_{n},f_{l}\right){|}^{2}}{\sum_{l=1}^{L}\sum_{m=1}^{M}a{\left(v_{n},f_{l}\right)}^{H}\Sigma_{I_{m}\left(f_{l}\right)}^{-1}a\left(v_{n},f_{l}\right)}\right)$$

Assuming that the noise is a wide smooth noise ${\sigma_{l}}^{2}=\sigma^{2}$ yields the following equation [25,27]:

(19) $${\hat{\sigma }}_{l}^{2}=\frac{tr[\left(I_{N}-P\right)R_{f}]}{M-1}$$

where *M* is the number of observation channels, $P=a_{n_{0}}{\left({a_{n_{0}}}^{H}a_{n_{0}}\right)}^{-1}{a_{n_{0}}}^{H}$, $a_{n_{0}}$ are the corresponding elements of the target velocity in the dictionary matrix, and $R_{f}=\sum_{m=1}^{M}I_{m}\left(f_{l}\right)I_{m}{\left(f_{l}\right)}^{H}/M$.

Let $\varepsilon_{\min}$ be the iteration termination threshold and the iteration termination condition be as follows:

(20) $$\varepsilon_{\min}\geq \frac{\left|\right|{\mathrm{fl}}^{k+1}-{\mathrm{fl}}^{k}|{|}_{1}}{|{\mathrm{fl}}^{k}{|}_{1}}$$

The result of the iteration fl is the output of the SBL-CS method.

## 3. Simulation Analysis and Test Dataset Processing

### 3.1. Simulation Analysis

**Figure 1:**
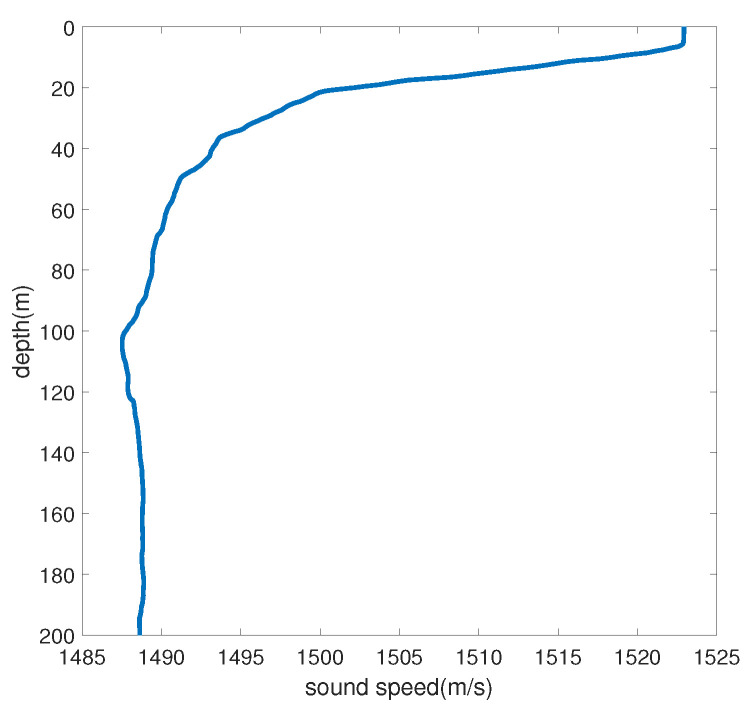
Gradient of sound velocity in simulation environment.

In this section, the target radial velocity estimation test is carried out using a simulation dataset, with a focus on analyzing the velocity estimation precision of the mutual spectrum method and the SBL-CS method. The simulation depth is 200 m, the underwater sound velocity is 1572.3 m/s, the density is 1.76 g/cm^3^, the attenuation coefficient is 0.2 dB/kmHz, and the hydrophone receiving depth is 50 m. The sound velocity gradient of the simulation environment is shown in Figure 1.

**Figure 2:**
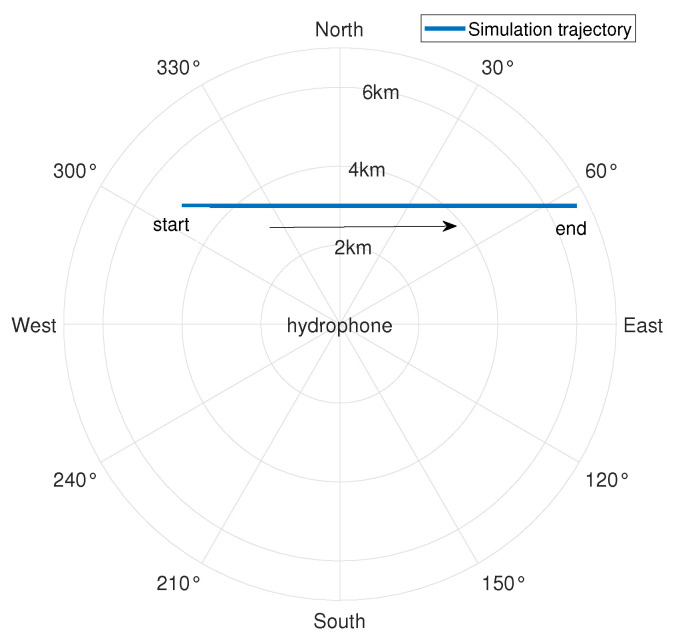
Simulation posture diagram.

**Figure 3:**
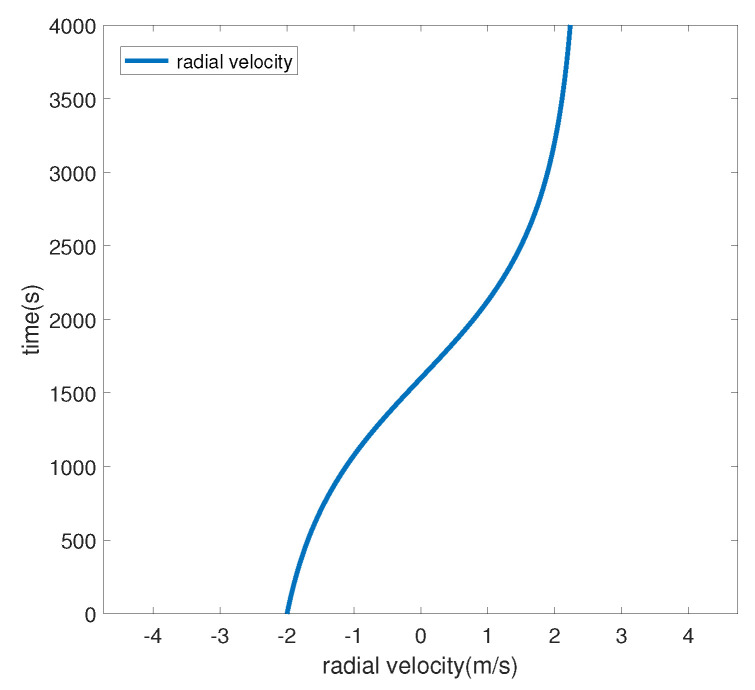
Simulated radial velocity diagram.

Taking the hydrophone as the origin and setting the surface target speed to 2.5 m/s, we set off at a position 5 km away from the hydrophone, with a heading angle of 90° (set the true north direction to 0° and increase the angle clockwise) and a nearest-passing distance of 3 km, voyaged for 4500 s. The target trajectory and the target radial velocity are shown in Figure 2 and Figure 3, respectively. A negative radial velocity value indicates that the target is approaching, while a positive value indicates that the target is moving away. The sound source emits 69, 117, 183, 259, and 315 Hz line spectrum signals.

**Figure 4:**
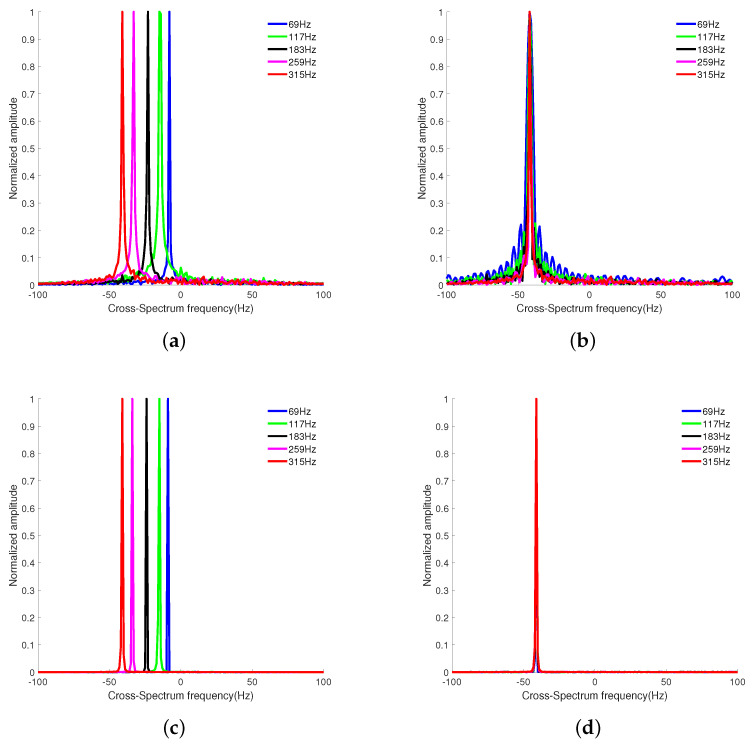
Plots showing the single−frequency processing results of the two methods; (**a**) Results of single−frequency processing of the CS method; (**b**) Compensated CS single−frequency processing results; (**c**) SBL-CS single−frequency processing results; (**d**) Compensated SBL−CS single−frequency processing results.

Set the SNR to 0 dB and the target sailing to 4000 s. The cross-correlation interval is taken as 0.5 s, the number of processing points is taken as 200 points, and the frequency of each frequency at the 0 moment is selected for solving to obtain the cross-spectrum sound intensity spectrum, as shown in Figure 4.

Figure 4a,c show that, if the phase is not compensated, the stripe frequencies solved for each frequency are not consistent and cannot be directly superimposed. Figure 4b,d demonstrate that the stripe frequencies of each frequency solution are consistent after phase compensation, with a target maximum line spectrum frequency of 315 Hz. Due to the low resolution of the original low-frequency solution results, the stripe frequency peaks of the 69 Hz and 117 Hz solutions in Figure 4 are wider and show obvious grating flaps. After the SBL-CS method phase compensates the dictionary matrix, the stripe frequencies of the solutions are consistent at each frequency and the grating flaps are suppressed. The spectral peaks calculated at each frequency are narrower and without obvious side flaps.

The SBL-CS method can not only utilize single-frequency point processing to suppress background noise and side flaps, but can also utilize multi-frequency fusion processing to further play its role in suppressing background noise.

**Figure 5:**
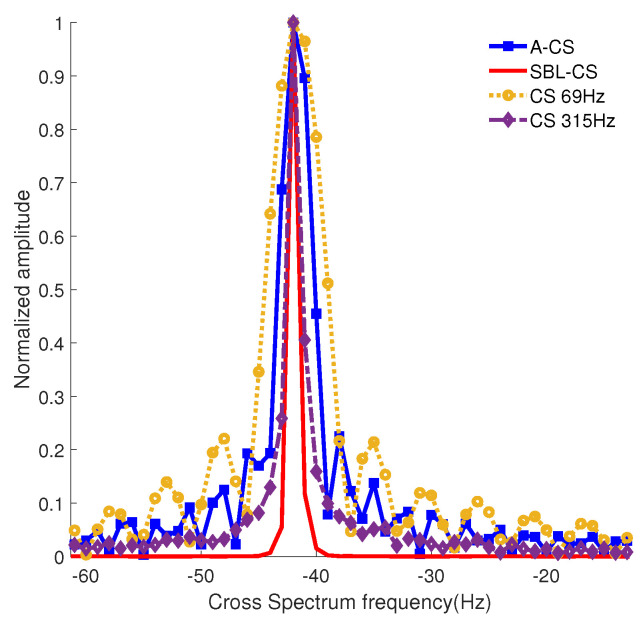
Multi−frequency processing results graph.

The results of the CS method, the spectrum accumulation CS method (A-CS), and the SBL-CS multi-frequency fusion processing method are shown in Figure 5. It can be seen that, due to the wider spectral peaks of the low-frequency (69 Hz) CS solution, the spectral peaks of the A-CS are also wider, so the CS method has a wider width of the main flap after spectral summing compared with the SBL-CS method. The SBL-CS solution not only obtains a narrower spectral peak width, but also effectively suppresses the background interference, which is more conducive to the extraction of the target velocity.

**Figure 6:**
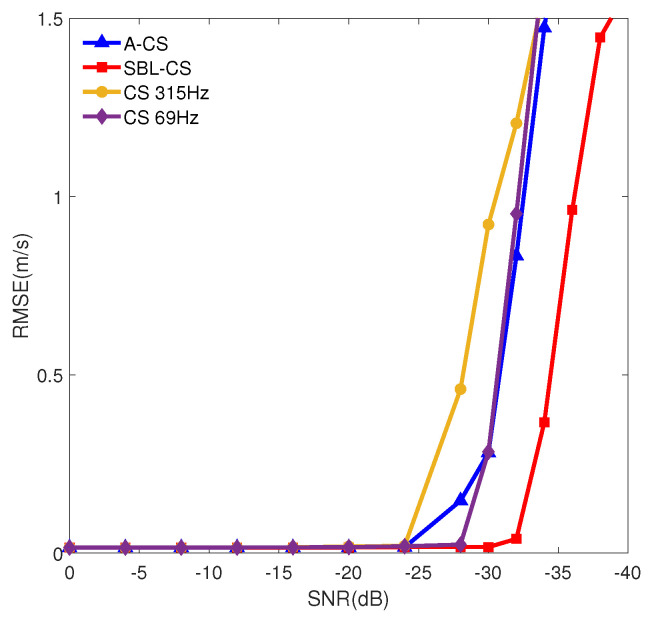
Comparison of the accuracy of different methods.

For the CS algorithm, there are significant differences in accuracy when processing at different frequencies. As shown in Figure 6, the CS algorithm has a significant difference in calculation accuracy between 69Hz and 315Hz at low signal-to-noise ratios, which is due to the low-speed resolution of low-frequency calculation results. The A-CS algorithm aligns and accumulates five frequencies.The results obtained show significant progress compared to the CS algorithm. Its performance declines more slowly compared to the CS algorithm, but its accuracy does not significantly improve at low signal-to-noise ratios. Compared with the A-CS algorithm, the SBL-CS algorithm can ensure slow performance degradation in low signal-to-noise ratio environments and withstand lower signal-to-noise ratio working environments. This indicates that SBL-CS is not simply a numerical addition, but enables effective data fusion, which further improves the performance of the algorithm.

**Figure 7:**
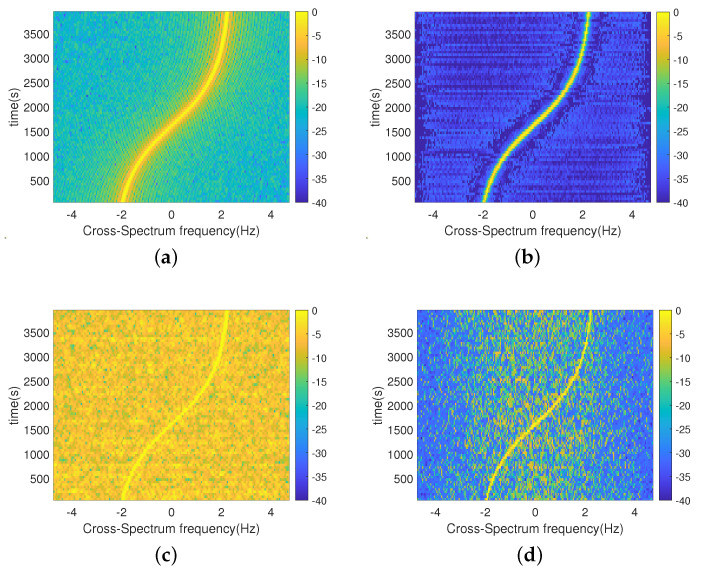
Plots showing single−frequency processing results of the two methods; (**a**) SNR = 0 dB; CS method results; (**b**) SNR = 0 dB; SBL−CS method results; (**c**) SNR = −30 dB; CS method results; (**d**) SNR = −30 dB; SBL−CS method results.

Figure 7 shows the simulation dataset processing results of the two methods, CS and SBL-CS, for different SNR conditions. Comparing Figure 7c,d, it can be seen that the CS method is more likely to be overwhelmed by the background noise, leading to method failure when the target velocity spectral peaks are under the condition of a low signal-to-noise ratio. Compared to Figure 7b,c, it can be seen that the SBL-CS method is able to suppress the background noise under low signal-to-noise ratio conditions.

In order to compare the performance of the two methods at different SNRs, a set of ablation experiments are designed to keep the simulation parameters unchanged and to vary and find the optimal SNR. As shown in Figure 6, 1000 Monte Carlo experiments are carried out at different SNRs, and the root mean square error of the velocity estimation is computed and obtained.

As can be seen from Figure 6, when the SNR is greater than −28 dB, there is no significant difference in the accuracy of the two methods. When the SNR is less than −28 dB, the performance of the CS method decreases rapidly. In contrast, the SBL-CS method shows a significant performance degradation only when the SNR is less than −32 dB, and the rate of performance degradation is slower compared to the CS method.

As can be seen from the combined Figure 6 and Figure 7, the SBL-CS method proposed in this paper is able to effectively suppress the environmental background noise and can work under lower signal-to-noise ratios. Furthermore, the method’s performance degradation rate is slower than that of the CS method.

### 3.2. Experimental Dataset Processing

**Figure 8:**
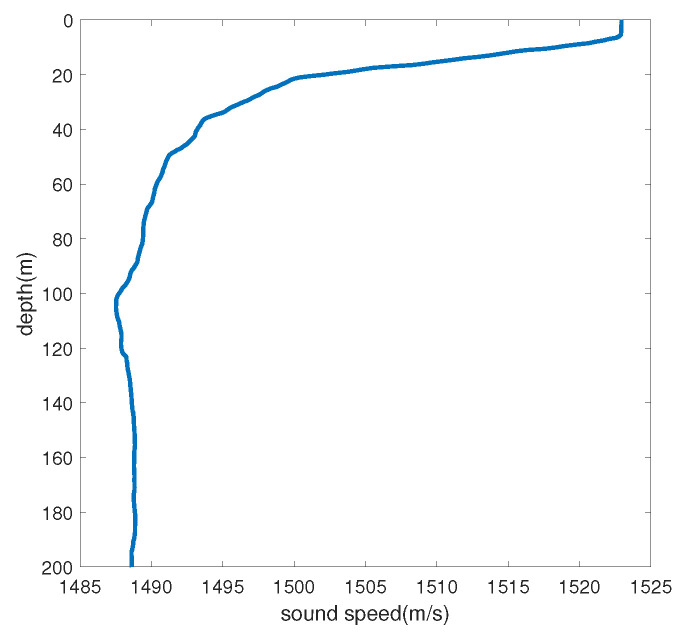
SWellEx−96 experimental sound velocity gradient.

**Figure 9:**
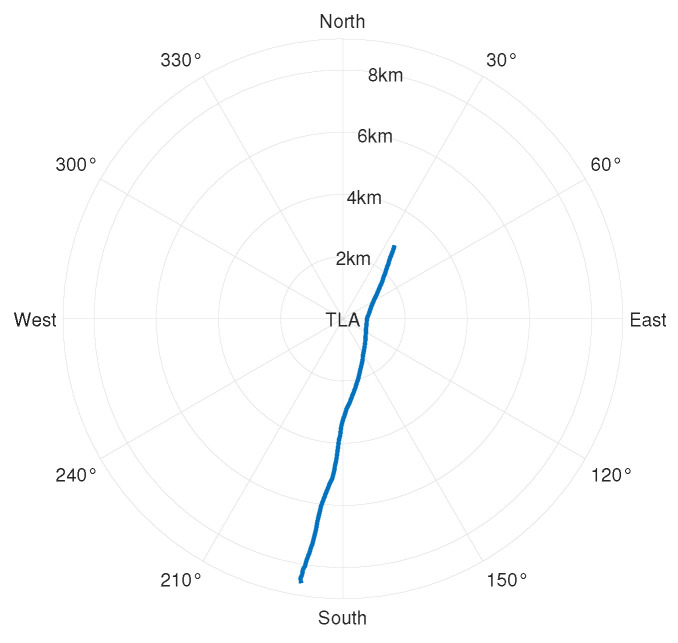
S5 incident posture map.

**Figure 10:**
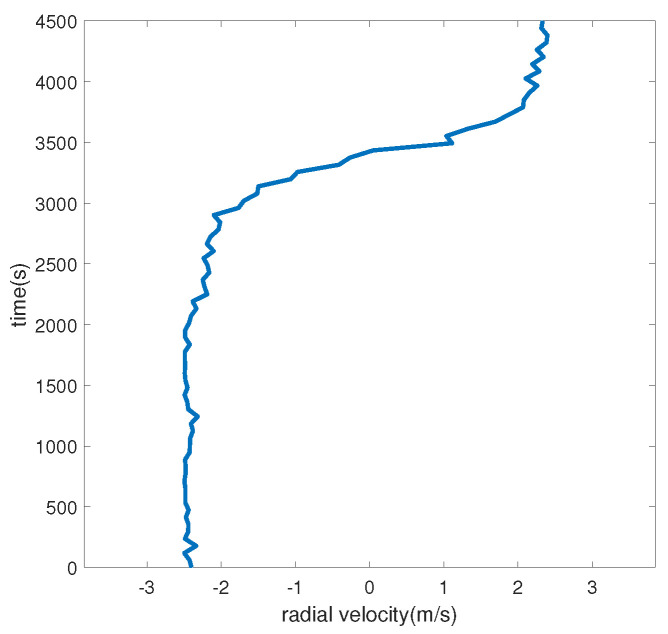
S5 event target radial velocity.

To verify the effectiveness of the method in a real environment, the S5 event in the SWellEx-96 experiment is selected for processing. The water depth of the experimental site is 212.5 m, the underwater sound speed is 1572.3 m/s, the density is 1.76 g/cm^3^, the attenuation coefficient is 0.2 dB/kmHz, and the sound speed gradient is shown in Figure 8. The sound source ship advances at a uniform speed of 5 knots, and the S5 event sound source ship trajectory and the radial velocity of the sound source ship are shown in Figure 9 and Figure 10, respectively. The sailing ship towed two sound sources at depths of 9 m and 54 m, respectively. The shallow source (at a depth of 9 m) emitted 109 Hz, 127 Hz, 145 Hz, 164 Hz, 198 Hz, 232 Hz, 280 Hz, 335 Hz, and 385 Hz frequency line spectra.

**Figure 11:**
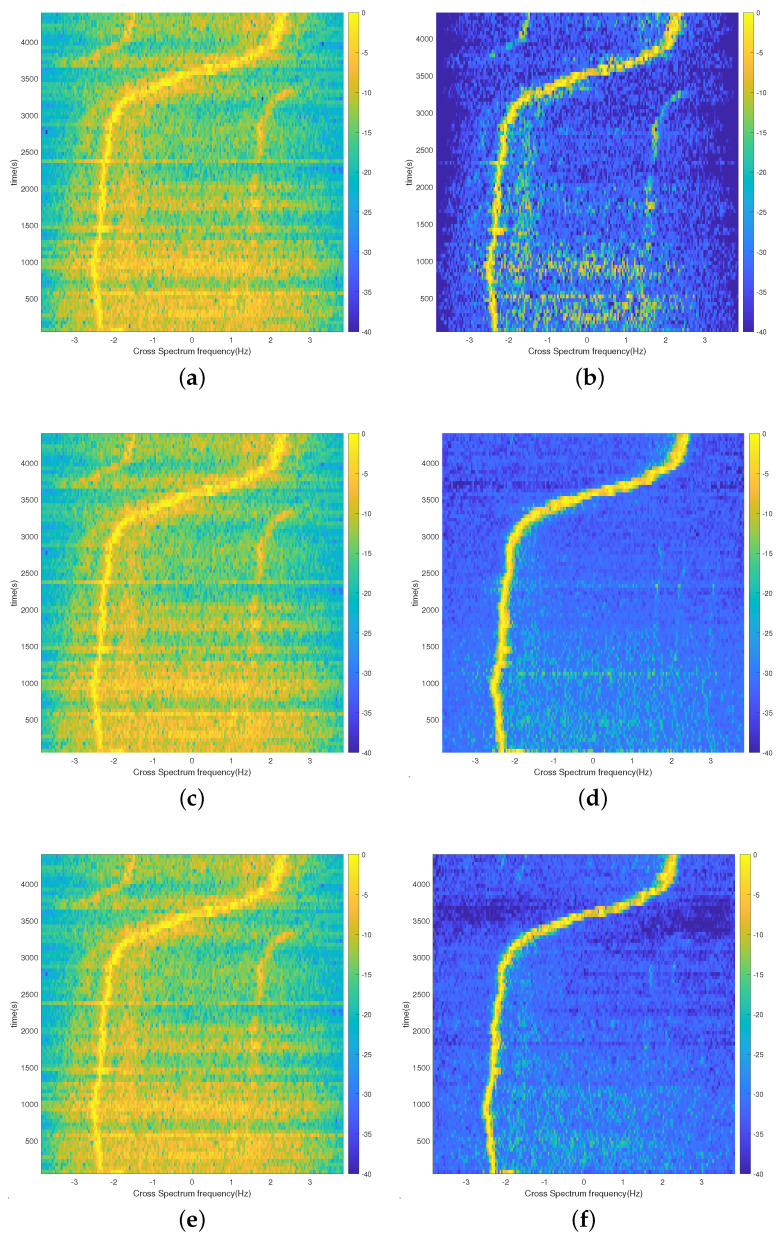
The S5 event-processing results for the three methods; (**a**) CS method; processing results at 385 Hz; (**b**) SBL-CS method; processing results at 385 Hz; (**c**) A-CS method; five-frequency-point processing results; (**d**) SBL-CS method; five-frequency-point processing results; (**e**) A-CS method; nine-frequency-point processing results; (**f**) SBL-CS method; nine-frequency-point processing results.

The second array element of the tilted line array(TLA) array is selected for processing, the inter-correlation interval is taken as 0.5 s, the number of processing points is 200 points in a single pass, and the frequency of solving the inter-spectral sound intensity is phase-compensated, with 385 Hz as the reference. To better verify the performance of the SBL-CS method, the 385 Hz single frequency, the selected five frequency points (127 Hz, 145 Hz, 232 Hz, 280 Hz, and 385 Hz), and all source frequencies (109 Hz, 127 Hz, 145 Hz, 164 Hz, 198 Hz, 232 Hz, 280 Hz, 335 Hz, and 385 Hz) were processed. The results of the 75 min dataset processing for the S5 event are shown in Figure 11. From the comparison of Figure 11a,b, the accuracy of the SBL-CS method is higher than the CS method in single-frequency processing and the background noise is suppressed. Figure 11c–f together illustrate that the A-CS method shows some improvement in accuracy in multi-frequency processing because of the direct addition of the inter-spectral results of different frequency points, but this improvement is limited. The SBL-CS method fuses the results of all the frequency points in the iterative process, so the more fused frequency points there are, the more the background noise suppression is obvious, and the more the accuracy is improved.

The RMSEs of the three methods are calculated for the S5 event, as shown in Table 1. The RMSE of the CS method is 0.5217 m/s and the RMSE of the SBL-CS method is 0.4549 m/s for single-frequency point processing in the S5 event. The RMSE of the A-CS method is 0.5180 m/s and that of the SBL-CS method is 0.3819 m/s for five-frequency-point processing. The error of the SBL-CS method is reduced by approximately 35.6%, compared with the A-CS method. For the processing of nine frequency points of the sound source, the RMSE of the A-CS method is 0.5180 m/s, while the RMSE of the SBL-CS method is 0.3545 m/s.The error of the SBL-CS method is reduced by about 46.1% compared with the A-CS method. This is consistent with the results of the simulation experiment. Although the A-CS algorithm aligns with the results of different frequency solutions, the gain obtained by direct accumulation is limited, resulting in limited accuracy improvement. The SBL-CS algorithm not only aligns the solution results of each frequency, but also effectively fuses the solution results, ultimately enabling the algorithm to maintain good accuracy under reduced signal-to-noise ratio conditions and to work normally in lower signal-to-noise ratio environments.

## 4. Conclusions

This paper aims to solve the problem of the CS method showing poor performance or even failing under low signal-to-noise ratio conditions when measuring the passive speed of hydroacoustic targets by fusing the SBL and the CS method, thereby putting forward the SBL-CS speed-measurement method for passive targets. The method realizes the fusion processing of different-frequency cross-spectrum results, and proves the feasibility and effectiveness of the SBL-CS method through simulation tests and the use of an actual dataset.

Comparing the SBL-CS method with the CS method, the following conclusions can be drawn. After compensation, the SBL-CS method is able to integrate the cross-correlating sound intensity of the multi-frequency to estimate the target velocity. The SBL-CS method is also able to suppress the background noise so that the peaks of the cross-correlating sound intensity, which carries the velocity information, are more prominent. When multi-frequency processing is carried out, the SBL-CS method integrates the multi-frequency estimates and is able to further suppress the background noise. Compared with the CS method, the SBL-CS method is able to work under lower signal-to-noise ratio conditions.

## Figures and Tables

**Table 1:** Comparison of the root mean square errors of the methods.

Algorithm	Frequency (Hz)	RMSE (m/s)
CS	385	0.5217
A-CS	127, 145, 232, 280, 385	0.518
	109, 127, 145, 164, 198, 232, 280, 335, 385	0.518
SBL-CS	385	0.4549
	127, 145, 232, 280, 385	0.3819
	109, 127, 145, 164, 198, 232, 280, 335, 385	0.3545

## Data Availability

The original contributions presented in the study are included in the article, further inquiries can be directed to the corresponding author.
